# Experimental and Numerical Investigation of the Effect of Projectile Nose Shape on the Deformation and Energy Dissipation Mechanisms of the Ultra-High Molecular Weight Polyethylene (UHMWPE) Composite

**DOI:** 10.3390/ma14154208

**Published:** 2021-07-28

**Authors:** Yonghua Shen, Yangwei Wang, Zhaopu Yan, Xingwang Cheng, Qunbo Fan, Fuchi Wang, Cheng Miao

**Affiliations:** 1 School of Materials Science and Engineering, Beijing Institute of Technology, Beijing 100081, China; shenyh1990@163.com (Y.S.); 3120191193@bit.edu.cn (Z.Y.); 2 National Key Laboratory of Science and Technology on Material under Shock and Impact, Beijing 100081, China; chengxw@bit.edu.cn (X.C.); fanqunbo@bit.edu.cn (Q.F.); wangfuchi@bit.edu.cn (F.W.); 3 Beijing Institute of Technology Chongqing Innovation Center, Chongqing 401147, China; 4 Inner Mongolia Metal Material Research Institute, Yantai 264003, China; miaocheng1983@163.com

**Keywords:** projectile nose shape, deformation mechanism, UHMWPE composite, specific energy absorption, ballistic resistance

## Abstract

The effect of projectile nose shape on the ballistic performance of the ultra-high molecular weight polyethylene (UHMWPE) composite was studied through experiments and simulations. Eight projectiles such as conical, flat, hemispherical, and ogival nose projectiles were used in this study. The deformation process, failure mechanisms, and the specific energy absorption (SEA) ability were systematically investigated for analyzing the ballistic responses on the projectile and the UHMWPE composite. The results showed that the projectile nose shape could invoke different penetration mechanisms on the composite. The sharper nose projectile tended to shear through the laminate, causing localized damage zone on the composite. For the blunt nose projectile penetration, the primary deformation features were the combination of shear plugging, tensile deformation, and large area delamination. The maximum value of specific energy absorption (SEA) was 290 J/(kg/m^2^) for the flat nose projectile penetration, about 3.8 times higher than that for the 30° conical nose projectile. Furthermore, a ballistic resistance analytical model was built based on the cavity expansion theory to predict the energy absorption ability of the UHMWPE composite. The model exhibited a good match between the ballistic resistance curves in simulations with the SEA ability of the UHMWPE composite in experiments.

## 1. Introduction

Fiber-reinforced plastic composites have been extensively used in armor protection due to low density and effective ballistic performance [1,2,3,4]. For the sake of the armor design, the parameter study of the ballistic impact has attracted intensive attention. These parameters include the mechanical property and structure of the composites [1,5], impact velocity, and projectile geometry [6,7,8]. The studies have found that the projectile shape has significant influence on the deformation and failure mechanisms the composites [9,10,11].

Relevant research demonstrates that the projectile shape can invoke different deformation and failure mechanism of the fiber-reinforced plastic composite. The majority of studies focus on the aramid fiber reinforced polymer (AFRP), glass fiber reinforced polymer (GFRP), and carbon fiber reinforced polymer (CFRP) composites [12,13]. Ulven [14] found that the conical shaped projectile resulted in the highest ballistic limit velocity, followed by the flat, hemispherical shaped projectiles. The primary failure modes of composites were a combination of the shear plugging and the separation of fibers. However, Millán [15] found that the effect of projectile shape on the ballistic limit of AFRP composite depended on the composite thickness. Jordan [16] revealed that the ballistic limit and energy absorption ability of the GFRP composite were significantly affected by the projectile nose shape. The results showed that the sharper nose shapes were the most efficient penetrators, and the flat nose projectiles exhibited the lowest penetration ability. In addition, the investigation also revealed that penetration of thinner targets was not influenced as much by the projectile nose shape [15]. Mitrevski [17] found that the blunter hemispherical impactor produced the largest damage area, while sharper conical impactor could produce a localized damage area.

Wen [18,19] proposed an analytical model for predicting the ballistic limit velocity of the composites impacted with various shaped projectiles. Ben-Dor [20] developed Wen’s model and found that the ballistic responses varied with the properties and thickness of the composites and the projectile nose shape. Tan [21] and Gibbon [22] confirmed the conclusions discovered by Ben-Dor [20] and found that the sharper nose projectile tended to push fibers aside as they penetrated through the composite. For the blunt nose projectile penetration, the primary deformation features consisted of the shear plugging and tensile deformation. Moreover, Ulven [14], Jordan [16], and Sasikumar [23] found that there was a high deviation between the predicted ballistic limit velocity and the experimental values, and they simultaneously pointed out that the accuracy of the analytical model was depended on the failure modes of the composite. The current analytical model was more suitable for the composites with local and shear failure mode damage [24].

Compared with other composites, the ballistic efficiency of the UHMWPE composite is found to be at least 30% higher than Kevlar^®^ KM2/polyvinyl butyral, 50% higher than E-glass/polyester composite, and 60% higher than carbon fiber/epoxy [25]. The unique mechanical property makes the UHMWPE composite exhibit different deformation and failure process [25,26,27]. The high fiber failure strain make the UHMWPE composite absorb the energy though large area tensile deformation [27,28]. The current studies reveal that the damage of the UHMWPE composite is a progressive deformation process containing the shear plugging, delamination, and tensile deformation [29,30,31,32]. However, few studies focus on the effect of projectile nose shape on the deformation features, penetration mechanisms, and energy absorption ability.

The current study mainly investigates the effect of projectile nose shape on the deformation and energy dissipation mechanisms of the UHMWPE composite. Eight projectiles such as conical, flat, hemispherical, and ogival nose projectiles are used in this study. The effect of projectile nose shape on the target responses of the deformation evolution, failure mechanisms, and energy absorption ability of the UHMWPE composite, and the residual velocity of the projectile were analyzed by the experimental and numerical analysis. Furthermore, the ballistic resistance analytical model was built based on the cavity expansion theory to predict the energy absorption ability of the UHMWPE composite. The current work can provide more understanding of the penetration and failure mechanism of the UHMWPE composite, which is meaningful guidance on the use of UHMWPE composite for specific protection needs.

## 2. Materials and Methods

### 2.1. UHMWPE Composite

In this study, the ballistic tests were conducted on the WT-120 UHMWPE composite, a unidirectional laminate produced by Beijing Protech New Material Science Co., Ltd. (Beijing, China). The laminate was stacked in a (0/90)n sequence by a hot pressing process under the pressure of 13 MPa and the temperature of 135 °C. The in-plane dimension of the UHMWPE laminate was 300 mm × 300 mm, and the thickness was approximately 20 mm.

### 2.2. The Projectile Material and Nose Shapes

The projectile material was 40CrMnSiB steel treated under the process of 870 °C quenching and 450 °C tempering heat treatment. The mechanical properties were summarized in Table 1. The projectile material possessed yield strength of 1350 MPa, elongation of 13.5%, and hardness of HRC45 ± 3.

**Figure 1:**
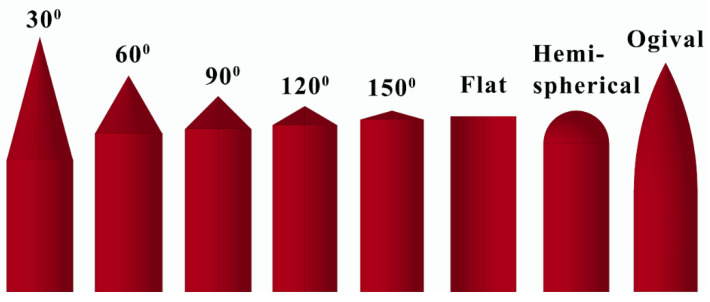
The projectile nose shapes in the experimental and numerical tests.

Figure 1 shows the projectile nose shapes in the experimental and numerical tests. All the projectiles had a diameter of 15 mm and a mass of 54 g. The projectile nose shape contained conical, flat, hemispherical, and ogival. Table 2 shows the geometrical parameters of the projectiles, for the conical nose projectiles, the nose length (L_N_), and the nose length-diameter (L_N_/D) decreased with the increasing of the nose angle, indicating the projectile become blunter. For the hemispherical nose projectile, the L_N_ and the L_N_/D were the same as those of the 90° conical nose projectile.

### 2.3. Ballistic Tests

**Figure 2:**
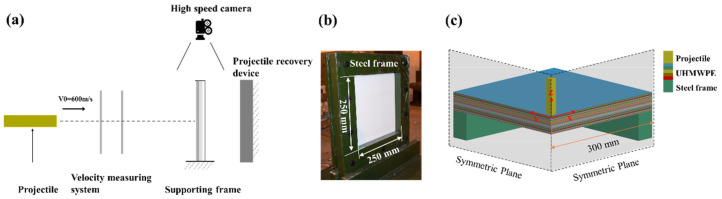
The tests process and the simulation model: (**a**) schematic diagram of the ballistic tests; (**b**) the steel frame for fixing the laminate; (**c**) the quarter-model for the impact configuration.

The ballistic performance of the UHMWPE composite was analyzed by conducting normal impact tests under a velocity of about 600 m/s. The projectiles were launched by the ballistic gun with a 25 mm caliber. Figure 2a shows the schematic diagram of the ballistic tests. The UHMWPE laminate was fixed by two steel frames, leaving a free deformation area of 250 mm × 250 mm, as shown in Figure 2b. The ballistic tests were carried out at Inner Mongolia Metal Material Research Institute (Yantai, Shandong Province, China). The high-speed camera was used to capture the deformation process of the UHMWPE composite and the residual velocity of the projectile. Moreover, the interior damage of the laminate was checked by the Direct Radiography (DR) non-destructive testing technique.

### 2.4. Numerical Simulations

To better explore the effect of the projectile nose shape on the ballistic response of the laminate, the ANSYS/LS-DYNA software was used to simulate the nonlinear high strain rate impact process. In this study, the Johnson–Cook (J–C) material model was used to describe the projectile and the steel frame [33,34]. The Mat-Composite-Damage (MCD) material model based on Chang–Chang damage criterion was adopted to describe the orthogonal anisotropic UHMWPE composite [35]. The Chang–Chang damage criterion included four failure models of the transverse tensile and compressive failure, the in-plane shear failure, and the delamination of the UHMWPE composite [36]. The effective plastic strain was set as 1.50 to control the deletion of the elements. The detailed material model parameters were listed in Table 3 and Table 4. A solid 164 hexahedron unit was adopted in the simulations, and the size of all the elements was 0.50 mm. The quarter three-dimensional (3D) models were built to improve the computational efficiency, as shown in Figure 2c. The UHMWPE laminate was divided into 40 layers, and the thickness of each layer was 0.5 mm.

## 3. Results and Discussion

### 3.1. The Deformation Process

**Figure 3:**
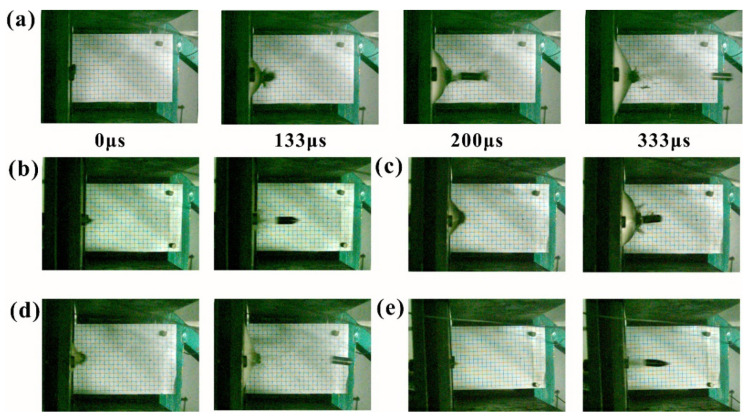
The deformation process of the UHMWPE laminate impacted various projectile nose shapes. (**a**–**e**) were flat, 60°, 120°, hemispherical, and ogival nose shape projectile, respectively.

With the help of the high-speed camera, the deformation process of the laminate and the projectile position was clearly observed, as shown in Figure 3. As the time interval of each frame was 66.67 μs, then the residual velocity of the projectile could be obtained through the travelled distance divided by the time. The front face of the composite was not observed by the high-speed camera, so the initial time of the projectile hitting the laminate was undefined. Herein, we set the previous frame of the visible deformation in the laminate as the initial time.

The deformation process of the UHMWPE laminate varied with the projectile nose shape, as shown in Figure 3. Figure 3a displays the deformation process of the UHMWPE laminate impacted with the flat nose projectile. A typically back face bulge was observed during the penetration process. The projectile penetrated into the laminate at 133 μs, and the back face bulge deformation (BD) and the diameter of the transverse deformation area (DTD) were 50 mm and 130 mm, respectively. However, the laminate deformation varied greatly after the direct penetration period, as shown in Figure 3a. The values of BD and DTD increased to 70 mm and 250 mm at 333 μs, respectively, which were 40% and 92% higher than those at the time when the projectile just penetrated into the laminate.

Figure 3b–e shows the deformation characteristics of the UHMWPE composite impacted with the 60°, 120°, hemispherical, and ogival nose shape projectiles, respectively. For the sharper 60° and ogival nose projectile penetration, the deformation of the laminate in Figure 3b,e was negligible. However, the back face bulge was obviously observed in Figure 3c,d. For the 120° conical projectile, the values of BD and DTD were 40 mm and 200 mm, respectively, which was close to those of the hemispherical projectile of 30 mm and 150 mm, respectively.

**Figure 4:**
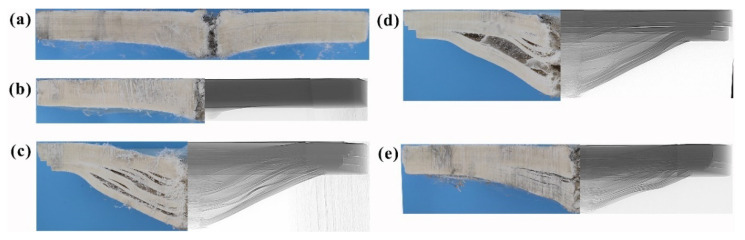
The deformation features of the UHMWPE laminate impacted various projectile nose shape. (**a**–**e**) were 60°, ogival, 120°, flat, and hemispherical nose shape projectile, respectively.

Figure 4 shows the deformation features of the UHMWPE laminate impacted with various nose shape projectiles. The left side was the macro-profile feature, while the right side was the interior damage detected by the DR method. For the sharper 60° and ogival nose projectile penetration, the laminate showed a completely shear failure, and the damage was limited to a small zone, as shown in Figure 4a,b. For the 120° conical and flat nose projectiles, the laminate deformation increased as the projectile nose became blunt, and the laminate experienced large tensile deformation and distinct delamination. Furthermore, the back face deformation area covered the whole plane, as shown in Figure 4c,d. Under the penetration process of the hemispherical nose projectile, the laminate exhibited moderate deformation, and Figure 4e showed that the deformation values of the BD and DTD were 30 mm and 150 mm, respectively. Therefore, the projectile nose shape had a dramatic influence on the deformation process of the UHMWPE composite.

### 3.2. Numerical Results

#### 3.2.1. The SEA Ability

In the ballistic tests, the residual velocity of the projectile was measured by the high-speed camera. Table 5 shows the residual velocity of the projectiles in experiments and simulations. The maximum relative error was 11.7%, indicating that the numerical results had a good agreement with the experimental results. For comparison purposes, the initial velocity of the projectile in simulations was set as 600 m/s. Previous studies discovered that the energy efficiency of shear failure model was lower than that of the tensile failure model in the UHMWPE composite [25,28]. Therefore, according to the deformation features of the laminate shown in Figure 4, a conclusion could be drawn that the penetration ability of the projectile from large to small was ogival, 60°, 120°, hemispherical, and flat nose projectile, successively.

The numerical results were completely consistent with the experimental results, as shown in Table 5. The residual velocity of the projectile decreased with the increase of projectile nose angle. When the nose angle was 30°, the residual velocity was 550m/s, suggesting that the UHMWPE composite had lower ballistic resistance. As the nose angle increased to 180° (i.e., flat nose), the residual velocity was decreased to 380 m/s, which was corresponded to the deformation characteristics of the laminate. In addition, the residual velocity of the hemispherical and ogival nose shape projectile was 480 m/s and 568 m/s, respectively.

**Figure 5:**
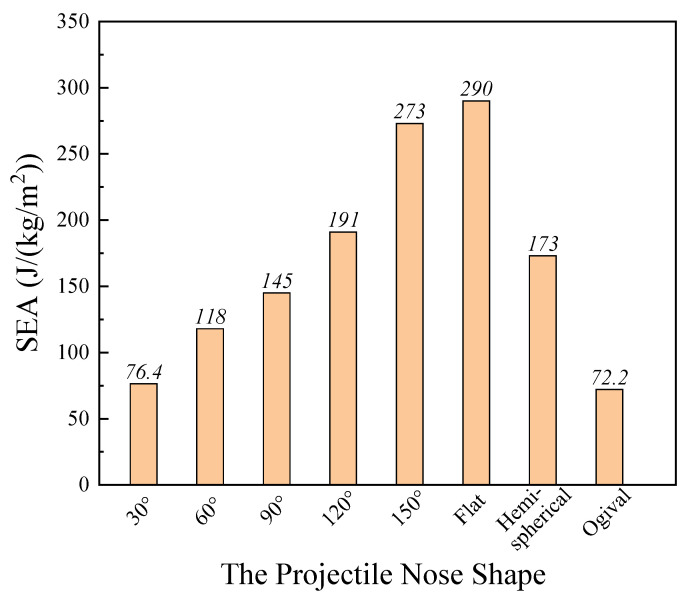
The specific energy absorption (SEA) of the UHMWPE composite impacted various nose shape projectiles.

To elaborate an underlying penetration mechanism, the effect of projectile nose shape on the specific energy absorption (SEA) of the laminate was further analyzed, as shown in Figure 5. The SEA corresponded to the deformation features of the laminate. For the conical nose shape projectile, when the projectile nose angle was in the range of 30° to 150°, the SEA values increased largely with the increase of the nose angle. However, the further increase of nose angle had little improvement on the SEA. The SEA increased only 6.2% when the projectile nose angle increased from 150° to 180°. The SEA value of composite under the 30° conical projectile penetration was minimum, 76.4 J/(kg/m^2^), while that of the 180° conical projectile penetration was maximum, 290 J/(kg/m^2^). In addition, under the penetration of the hemispherical and ogival projectiles, the values of SEA were 173 J/(kg/m^2^) and 72.2 J/(kg/m^2^), respectively.

#### 3.2.2. Projectile Responses

**Figure 6:**
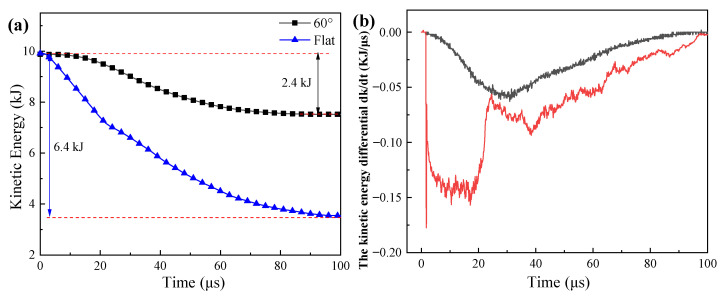
The ballistic responses of the projectile during the penetration process: (**a**) the kinetic energy curves of the projectile; (**b**) the differential curves of kinetic energy, *dk*/*dt*.

**Figure 7:**
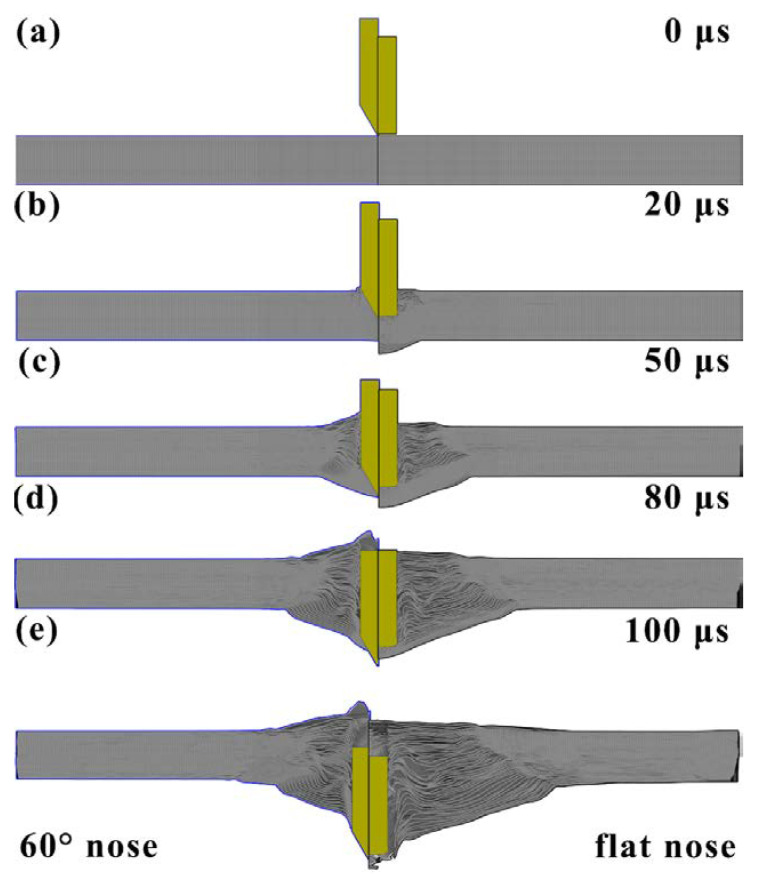
The damage evolution of the laminate impacted with 60° and flat nose projectile: (**a**) for the 60° conical and flat nose projectiles penetration; (**b**) the projectile nose penetrated into the composite; (**c**) the bulge deformation and delamination of the fibers; (**d**) the tensile deformation of the composite; (**e**) the damage morphology after the penetration.

Taking the case of the 60° conical and flat nose projectile, the effect of projectile nose shape on the ballistic responses of the projectile was analyzed, as shown in Figure 6. Under the penetration of 60° nose projectile, the laminate consumed 2.4 kJ kinetic energy, much less than that of the flat nose projectile of 6.4 kJ, as shown in Figure 6a. The parameter *k* represented the kinetic energy of the projectile, the kinetic energy differential curves (*dk*/*dt*) in Figure 6b represented the changing rate of the kinetic energy of the projectile. Moreover, Figure 7 shows the damage evolution of the laminate impacted with 60° and a flat nose projectile.

When the 60° nose projectile penetrated the UHMWPE composite, the projectile nose shape was sharp and high enough to penetrate through the composite easily, as shown in Figure 7a. Due to the increase of contact area between the projectile and the laminate, the kinetic energy differential curves increased in the first 25 μs, and the maximum value was about 0.06 kJ/μs, as shown in Figure 6b. After that, the projectile began to penetrate the composite, as shown in Figure 7b. The fibers were pushed aside and the ballistic resistance decreased, as the dk/dt curve shown in Figure 6b. The damage was limited to a small zone, as shown in Figure 7d,e.

Compared with the 60° conical nose projectile, Figure 6b showed that there were significantly different variation rules in the kinetic energy differential curve of the flat nose projectile. Due to the larger contact area, the *dk*/*dt* curve increased rapidly and then remained steady. In the first 20 μs, the value the *dk*/*dt* was about 0.15 kJ/μs, much higher than that of 60° nose projectile, as shown in Figure 6b. As shown in Figure 7b, many fibers were in compressive conditions, resulting in the small back face bulge deformation. Afterward, the failure mechanisms of the UHMWPE composite transformed from the shear deformation to the tensile deformation, resulting in a small decrease of the ballistic resistance. Finally, the remaining laminate consumed the kinetic energy of the projectile through tensile deformation, causing the large area of laminate delamination, as shown in Figure 7c. Although the ballistic resistance was decreasing, the duration of the tensile deformation stage was the longest. Figure 6b shows that the changing rate of the kinetic energy of the projectile increased between 25–40 μs, and then decreased gradually after 40 μs. With the development of the penetration process, the thickness of the remaining laminate became thinner, and the tensile deformation and the transverse deformation area, increased gradually, as shown in Figure 7d,e. When the flat nose projectile penetrated though the laminate, the values of BD and DTD were 50 and 140 mm, respectively, which was consistent with the result (133 μs) in Figure 3a. As observed by the high-speed camera in Figure 3a, the deformation of the composite would increase continuously.

#### 3.2.3. The Deformation Characteristics

**Figure 8:**
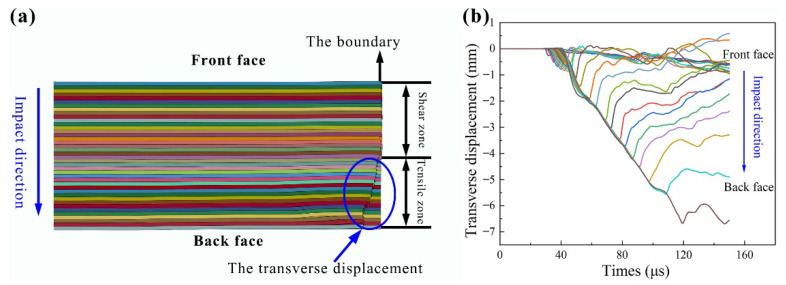
The transverse displacement of the laminate in simulations: (**a**) the shear and tensile zone, (**b**) the transverse displacement history curves of each layer.

In the experiments and simulations, the tensile deformation of the laminate made the fibers move lateral, resulting in the slip step in the boundary, as shown in Figure 4 and Figure 8a. Herein, according to the failure model of the laminate, the whole laminate was divided into the shear zone and the tensile zone. The thickness of the shear and tensile zones were determined by the transverse displacement of the boundary elements. Figure 8b shows the transverse displacement history curves of the boundary elements in each layer, corresponding to the laminate deformation in Figure 8a. In the shear zone, the damage was limited to the penetration zone, resulting in a small transverse displacement. While in the tensile zone, the transverse displacement increased gradually, as shown in Figure 8b, and the maximum value was about 7.0 mm.

**Figure 9:**
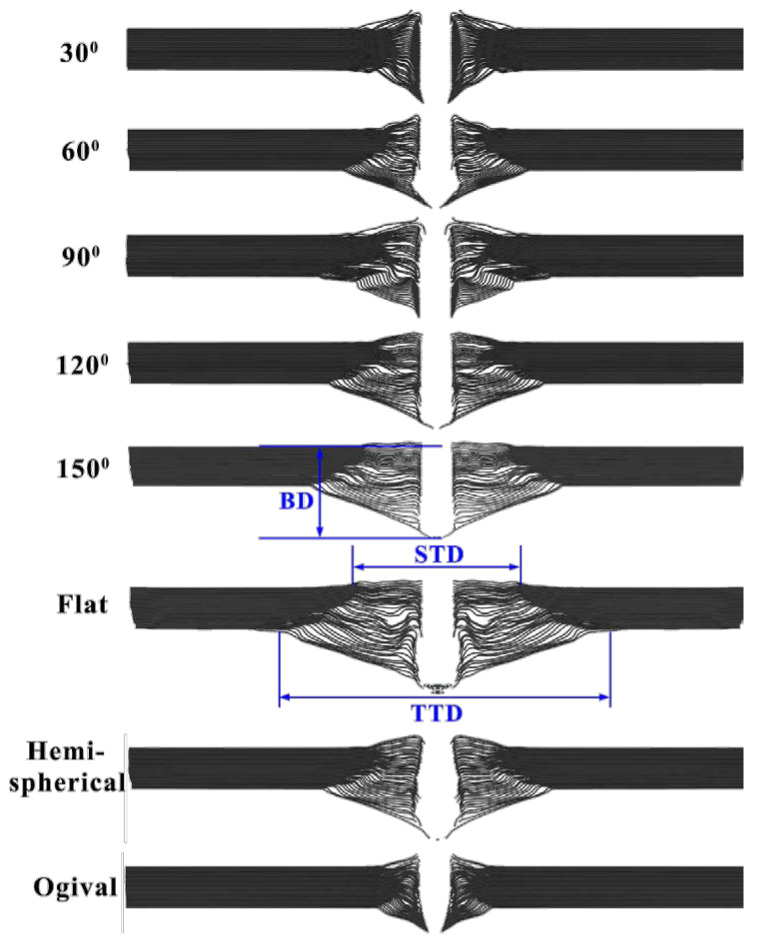
The macro-profile features of the UHMWPE composite impacted with various nose shape projectile.

To further elucidate the penetration and failure mechanisms, the macro-profile feature of the UHMWPE composite was analyzed, as shown in Figure 9. The shear zone thickness (defined as ST, the same below) and tensile zone thickness (TD), the diameter of the transverse deformation area of shear zone (STD), the diameter of the transverse deformation area of tensile zone (TTD) and the back face bulge deformation (BD) were measured to describe the deformation characteristics of the UHMWPE composite.

Table 6 shows the deformation parameters of the UHMWPE composite in simulations. For the conical nose projectile, when the conical nose angle was less than 120°, due to the obconical nose shape, the contact area between the projectile nose and the laminate increased until the whole projectile nose penetrated the laminate. The fibers below the projectile nose were pushed laterally, causing a distinct bulge in the front face, as shown in Figure 9. The damage was limited in the impact zone, and the back face bulge deformation was small.

With the increase of the conical angle, the length of the projectile nose decreased, resulting in the change of the penetration mechanism. The phenomenon of the fibers pushing aside was disappeared, and the tensile deformation of the laminate was more and more obvious. As shown in Table 6, the back face bulge deformation and the diameter of the transverse deformation area of tensile zone increased with the increase of the conical angle. For the 30° conical nose projectile, the value of the ST was 18 mm, indicating that almost all of the laminate was damaged under the shear stress. However, under the penetration of the flat nose projectile, the value of the ST decreased to 10 mm, and about 10 mm thick fibers experienced large tensile deformation. The values of BD and TTD were 52 and 170 mm, which were much larger than those in the 30° conical nose projectile.

The hemispherical nose projectile had a similar penetration mechanism with the large conical nose projectile. The values of TT, TTD, and BD were comparable with those of 120° conical nose projectile, which were 5 mm, 112 mm, and 42 mm, respectively, as shown in Table 6. In addition, owing to the similar nose shape, the laminates of the ogival nose and 30° conical nose projectiles had similar macro-profile features.

## 4. Analytical Model

Wen’s model [18,19] was widely used in the studies to analyze the effect of projectile nose shape on the ballistic limit of fiber-reinforced plastic (FRP) composites [14,16]. However, Wen’s model was more applicable for the FRP composite under the shear failure, rather than the composite under tensile failure [18,27]. Based on the cavity expansion theory [37], the ballistic resistance models with various nose shape projectiles were built in the present study.

**Figure 10:**
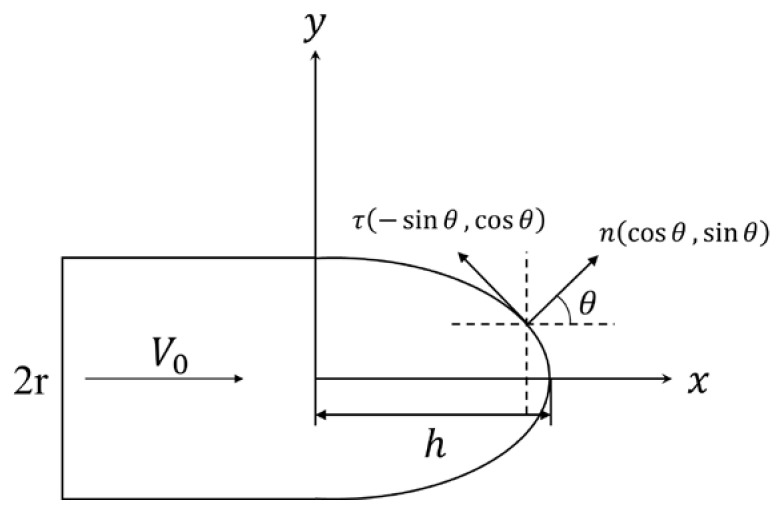
The typically projectile nose shape.

Due to the high hardness and strength of the projectile, the mass loss and deformation of the projectile were negligible during the penetration process. The projectile could be regarded as a rigid body. The typically projectile nose shape is shown in Figure 10; based on the cavity expansion theory, when the rigid projectile penetrated the UHMWPE composite, the ballistic resistance of the projectile was expressed as follows [37,38]:

(1) $$F=\pi r^{2}\left(AY_{t}N_{1}+B\rho_{t}V_{0}^{2}N_{2}\right)$$

Herein, the r was the radius of the projectile, A and B were the static and dynamic coefficients of frictional resistance, respectively. $\rho_{t}$ was the density of the UHMWPE composite, $V_{0}$ was the initial velocity of the projectile, and $N_{1}$ and $N_{2}$ were the coefficients of the projectile nose shape.

Typically, the projectile nose shape could be expressed by a bus function, and the coefficient of the projectile nose shape, $N_{1}$ and $N_{2}$, could be simplified to [39]:

(2) $$N_{1}=1+\frac{2\mu_{m}}{r^{2}}\int_{0}^{h}ydx$$

(3) $$N_{2}=N^{*}+\frac{2\mu_{m}}{r^{2}}\int_{0}^{h}\frac{yy^{{}^{\prime }2}}{1+y^{{}^{\prime }2}}dx$$

(4) $$N^{*}=\frac{2}{r^{2}}\int_{0}^{h}\frac{yy^{{}^{\prime }3}}{1+y^{{}^{\prime }2}}dx$$

**Figure 11:**
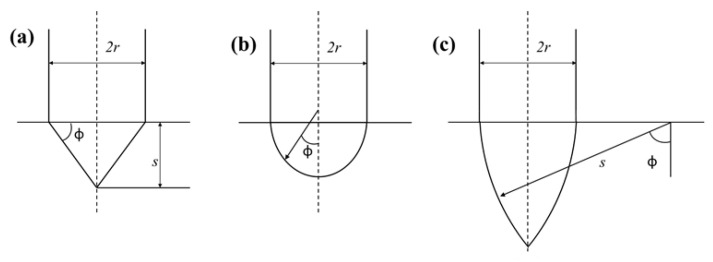
The projectile geometries with various nose shapes. (**a**) conical; (**b**) hemispherical; (**c**) ogival.

Herein, the non-dimensional parameter φ=s/(2r), s was the radius of the projectile nose, as shown in Figure 11. Therefore, for the conical nose shape projectile, the coefficient of the projectile nose shape, $N_{1}$, $N_{2}$ and $N^{*}$ could be further simplified as follows [39]:

(5) $$N_{1}=1+2\mu_{m}\varphi$$

(6) $$N_{2}=N^{*}+\frac{2\mu_{m}\varphi }{1+4\varphi^{2}}$$

(7) $$N^{*}=\frac{1}{1+4\varphi^{2}}$$

For the hemispherical nose shape projectile, the coefficients of the projectile nose shape, $N_{1}$, $N_{2}$, and $N^{*}$ could be further simplified as follows [39]:

(8) $$N_{1}=1+2\mu_{m}\varphi^{2}\left(2\varphi_{0}-\sin2\varphi_{0}\right)$$

(9) $$N_{2}=N^{*}+\mu_{m}\varphi^{2}\left(\varphi_{0}-\frac{\sin4\varphi_{0}}{4}\right)$$

(10) $$N^{*}=1-\frac{1}{8\varphi^{2}},\frac{1}{2}\leq N^{*}\leq 1$$

(11) $$\varphi_{0}=arc\sin\left(\frac{1}{2\varphi }\right),\varphi \geq \frac{1}{2}$$

For the ogival nose shape projectile, the coefficient of the projectile nose shape, $N_{1}$, $N_{2}$, and $N^{*}$ could be further simplified as follows [39]:

(12) $$N_{1}=1+4\mu_{m}\varphi^{2}\left[\left(\frac{\pi }{2}-\varphi_{0}\right)-\frac{\sin2\varphi_{0}}{2}\right]$$

(13) $$N_{2}=N^{*}+\mu_{m}\varphi^{2}\left[\left(\frac{\pi }{2}-\varphi_{0}\right)-\frac{1}{3}\left(2\sin2\varphi_{0}+\frac{\sin4\varphi_{0}}{4}\right)\right]$$

(14) $$N^{*}=\frac{1}{3\varphi }-\frac{1}{24\varphi^{2}},0\leq N^{*}\leq \frac{1}{2}$$

(15) $$\varphi_{0}=arc\sin\left(1-\frac{1}{2\varphi }\right),\varphi \geq \frac{1}{2}$$

Especially for the flat nose projectile, $N_{1}=N_{2}=1$, while for the hemispherical nose projectile, $N_{1}=1+\left(\mu_{m}\cdot \pi \right)/2$, $N_{2}=1/2+\left(\mu_{m}\cdot \pi \right)/8$, $N^{*}=1/2$. In the penetration process, the value of the $\mu_{m}$ was about 0.1 [39]; therefore, $N_{2}/N_{1}=N^{*}$. The ballistic resistance of the projectile Formula (1) could be simplified and expressed as follows:

For conical nose shape projectile:

(16) $$F=\pi r^{2}\left(AY_{t}+B\rho_{t}V_{0}^{2}\frac{1}{1+4\varphi^{2}}\right)$$

For hemispherical nose shape projectile:

(17) $$F=\pi r^{2}\left(AY_{t}+B\rho_{t}V_{0}^{2}\left(1-\frac{1}{8\varphi^{2}}\right)\right)$$

For ogival nose shape projectile:

(18) $$F=\pi r^{2}\left(AY_{t}+B\rho_{t}V_{0}^{2}\left(\frac{1}{3\varphi }-\frac{1}{24\varphi^{2}}\right)\right)$$

**Figure 12:**
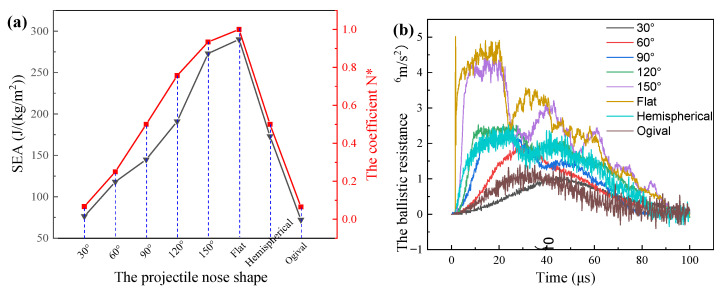
(**a**) The specific energy absorption of the UHMWPE composite under the penetration of various nose shape projectile and the coefficient of the projectile nose shape; (**b**) the ballistic resistance curves of projectiles.

In this study, the parameters, *πr*^2^, $AY_{t}$, and $B\rho_{t}V_{0}^{2}$ were constant, which were related to the projectile and UHMWPE composite themselves. Therefore, the ballistic resistance was proportionate to the coefficient of the projectile nose shape of N*. Firstly, the parameter s was measured, and then the parameters φ were calculated according to the formula φ=s/(2r). Finally, the parameters of N* were obtained according to the formulas of (7), (10), and (14), as shown in Table 7. The SEA ability of the UHMWPE composite directly reflected the ballistic resistance of the projectile. Figure 12a shows the effect of the projectile nose shape on the parameters of SEA and N*, and these two parameters had a similar changing trend. The parameter of SEA and N* increased with the increase of the conical nose angle. The maximum value of N* was 1.0 for the flat nose projectile. The coefficient of the ogival projectile was 0.065, which was approximately equal to that of the 30° conical nose projectile, resulting in the similar SEA ability of the UHMWPE composite.

To further analyze the effect of the projectile nose shape on the response of the projectile, the ballistic resistance curves of various projectiles were shown in Figure 12b. Obviously, the curves could be divided into three types, namely, the curves of 30°, 60°, and ogival projectiles, the curves of 90°, 120°, and hemispherical projectiles and the curves of 150° and flat. The 30°, 60°, and ogival nosed projectile possessed a high nose length of 28 mm, 13 mm, and 29 mm, respectively, as shown in Figure 1 and Table 2. In the first period, the projectile nose was sharp and high enough to penetrate through the UHMWPE composite easily. Due to the increase of contact area between the projectile and the composite, the ballistic resistance curves increased slowly. However, the different parameters *φ* made the contact area between the projectile and the composite slight differences, resulting in a difference in the slope and the amplitude value. In the second period, the projectile shoulder began to penetrate the composite, and the fibers were pushed aside and the ballistic resistance decreased.

The nose length of the 90°, 120°, and hemispherical projectiles was 7.5 mm, 4.3 mm, and 7.5 mm, respectively. Due to the lower nose length and parameters *φ*, the ballistic resistance increasing rate was larger than those of 30°, 60°, and ogival projectiles. The ballistic resistance remained steady as the projectile shoulder penetrated the composite. Afterwards, the failure mechanisms of the UHMWPE composite transformed from the shear deformation to the tensile deformation, resulting in a small decrease of the ballistic resistance. Finally, the fibers on the back face absorbed the kinetic energy of the projectile through large tensile deformation. Although the ballistic resistance was decreasing, the duration of the tensile deformation stage was the longest. Significantly, the 150° and flat ballistic resistance curves were similar with those of the 90°, 120°, and hemispherical, indicating the similar deformation process. However, the lower nose length and parameters of *φ* made a larger contact area than others, causing a higher ballistic resistance amplitude values, as shown in Figure 12b. In summary, the nose length and the parameters *φ* had a significant effect on the deformation mechanism of the composites and the ballistic resistance of the projectiles. Therefore, for the projectiles with different nose angle and shapes, when the length and the parameters of *φ* were comparable, the UHMWPE composite would exhibit similar deformation features and energy dissipation ability.

## 5. Conclusions

The projectile nose shape had a significant effect on the deformation and energy dissipation of the UHMWPE composite. For the sharper 30°, 60°, and ogival nose projectile penetration, the laminate showed a completely shear failure, and the damage was limited to a small zone, while for the 90°, 120°, and hemispherical nose projectile penetration, the pushing effect on the fibers diminished gradually, and the tensile deformation was more and more obvious. For the 150° and flat nose projectile penetration, the tensile deformation was predominant, and the back face deformation and delamination covered the whole plane.

The results showed that the sharper nose projectiles were the most efficient penetrators, and the flat nose projectiles exhibited the lowest penetration ability. The SEA ability of UHMWPE composite increased largely in a certain range; however, the further increase of projectile nose angle had little improvement on the SEA. The maximum SEA value of composite under the flat nose projectile penetration was 290 J/(kg/m^2^), about 3.8 times higher than that of 30° conical nose projectile penetration.

A ballistic resistance analytical model was built based on the cavity expansion theory to predict the energy absorption ability of the UHMWPE composite. The model exhibited a good match between the ballistic resistance curves in simulations and the specific energy absorption ability of a UHMWPE composite in experiments. Furthermore, when the length and the parameter *φ* of the projectile nose were comparable, the UHMWPE composite would exhibit similar deformation features and energy dissipation ability.

## Figures and Tables

**Table 1:** The mechanical properties of 40CrMnSiB steel.

Material	Hardness HRC	Young’s Modulus (GPa)	Yield Strength (MPa)	Tensile Strength (MPa)	Elongation (%)
40CrMnSiB	45 ± 3	200	1350	1558	13.5

**Table 2:** The geometrical parameters of the projectiles.

Projectiles	Length, L (mm)	Nose length, L_N_ (mm)	Diameter, D (mm)	L_N_/D
30° conical	58	28	15	1.867
60° conical	48.5	13	15	0.867
90° conical	44.5	7.5	15	0.500
120° conical	42.3	4.3	15	0.286
150° conical	41	2	15	0.133
Flat	40	0	15	0
Hemispherical	41.5	7.5	15	0.500
Ogival	52	29	15	1.933

**Table 3:** Johnson–Cook material model parameters for the projectile and the steel frame.

Parameters	Steel Frame	Projectile
Density, ρ (g/cm^3^)	7.80	7.80
Young’s modulus, G (GPa)	200	210
Yield stress, A (GPa)	1.18	1.50
Hardening constant, B (GPa)	0.17	0.20
Hardening exponent, n	0.28	1.555
Strain rate constant, C	0.058	0.058
Ref. strain rate, ${\dot{\varepsilon }}_{0}$ (s^−1^)	1.00	1.00
Thermal softening exponent, m	1.13	0.80
Melting temperature, (K)	1793	1773
Damage constant, D1	0.123	0.6
Damage constant, D2	0.00	3.04
Damage constant, D3	0.00	1.28
Damage constant, D4	0.694	0.10
Damage constant, D5	0.501	1.60

**Table 4:** MCD material model parameters for the UHMWPE composite.

Parameters	UHMWPE Composite
Density, ρ (g/cm^3^)	0.98
Young’s modulus, Ex (GPa)	26.6
Young’s modulus, Ey (GPa)	26.6
Young’s modulus, Ez (GPa)	2.60
Poisson ratio, $v_{yx}$	0
Poisson ratio, $v_{zx}$	0.008
Poisson ratio, $v_{zy}$	0.008
Shear modulus, Gxy	2.00
Shear modulus, Gyz	1.60
Shear modulus, Gzx	1.60
Shear strength, ab plane, SC (GPa)	0.56
Longitudinal tensile strength, XT (GPa)	1.90
Transverse tensile strength, YT (GPa)	1.90
Transverse compressive strength, YC (GPa)	1.20
Normal tensile strength, SN (GPa)	0.95
Transverse shear strength, Syz (GPa)	0.95
Transverse shear strength, Szx (GPa)	0.95

**Table 5:** The residual velocity of the projectiles in experiments and simulations.

Nose Shape	Experimental Results (m/s)		Simulational Results (m/s)		Error (%)
	Impact Velocity	Residual Velocity	Impact Velocity	Residual Velocity	
30°	/	/	600	550	/
60°	568	494	568	482	−2.20
			600	523	
90°	/	/	600	503	/
120°	580	376	580	420	11.70
			600	450	/
150°	/	/	600	401	/
Flat	600	400	600	380	−5.00
Hemispherical	566	430	566	438	1.86
			600	480	/
Ogival	596	576	600	568	2.7

**Table 6:** The deformation parameters of the UHMWPE composite in simulations.

Parameters	30°	60°	90°	120°	150°	Flat	Hemi-Spherical	Ogival
ST (mm)	18	17	16	15	14	10	15	20
STD (mm)	45	50	52	66	76	90	72	25
TT (mm)	2	3	4	5	6	10	5	0
TTD (mm)	45	80	90	102	125	170	112	25
BD (mm)	25	27	30	42	46	52	42	25

**Table 7:** The parameters of the projectile nose shape.

Parameters	30°	60°	90°	120°	150°	Flat	Hemi-Spherical	Ogival
s (mm)	28	13	7.5	4.3	2	0	7.5	70
*φ*	1.8667	0.8667	0.5000	0.2867	0.1333	0	0.5000	4.6667
N*	0.0670	0.2497	0.5000	0.7526	0.9336	1.0000	0.5000	0.0650

## Data Availability

Not applicable.
